# Supplementary material for: A Translational Model of MASLD-Associated HFpEF Defines Mitochondrial Dysfunction and Cardiac Plasticity During Disease Progression and Regression
Source: bioRxiv. 2026 Feb 28:2026.02.26.708088. Preprint. [Version 1] doi: 10.64898/2026.02.26.708088 (PMC13160009; doi:10.64898/2026.02.26.708088)
Supplement: Supplement 1 [file media-1.docx]

**A Translational Model of MASLD-Associated HFpEF Defines Mitochondrial Dysfunction and Cardiac Plasticity During Disease Progression and Regression**

Souradipta Ganguly^1,2^, Betul Gunes^2^, Yusu Gu^2^, Jorge Suarez^2^, Gautam Gupta^1^, Kei Ishizuka^2^_,_ Rabi Murad^1^, Tatiana Kisseleva^3^, Wolfgang Dillmann^2^, Kirk Peterson^2^, Eric Adler^2^, David A. Brenner^1,2#^, Debanjan Dhar^1,2#^

^1^Sanford Burnham Prebys Medical Discovery Institute, La Jolla, CA, USA

^2^Department of Medicine, University of California, San Diego, La Jolla, CA, USA

^3^Department of Surgery, School of Medicine, University of California, San Diego, CA, USA

^#^Correspondence: [ddhar@sbpdiscovery.org](mailto:ddhar@sbpdiscovery.org); [dbrenner@sbpdiscovery.org](mailto:dbrenner@sbpdiscovery.org)

**Running Title:** Cardiometabolic remodeling in MASLD

**Keywords:** HFpEF, Metabolic Dysfunction associated steatotic liver disease (MASLD), MASH, LV Dysfunction, Lifestyle Modification, Disease Regression, Liver-Heart Axis

**SUPPLEMENTARY METHODS**

**Isolation of Cardiac Tissue**

Cardiac tissue was collected following diastolic arrest unless otherwise noted. Briefly, mice were anesthetized with isoflurane, after which 0.3 M KCl was injected into the heart to induce rapid diastolic arrest. The heart was then excised and washed in 0.3 M KCl to remove residual blood. For experiments requiring left ventricular tissue, the arrested heart was transferred to ice-cold phosphate-buffered saline (PBS), and the left ventricle (LV) was identified and carefully dissected away from the right ventricle and interventricular septum. LV tissue was immediately processed for downstream applications.

**Histology and Image Quantification**

Tissue samples, including the left lateral liver lobe and whole heart, were fixed in 10% neutral-buffered formalin for 48 hours, followed by paraffin embedding and sectioning. Liver sections were stained with hematoxylin and eosin (H&E) and Picro Sirius Red to assess general morphology and collagen deposition, respectively. Cardiac sections were stained with Wheat Germ Agglutinin (WGA; RL-1022, Vector Laboratories) for delineation of cardiomyocyte size and immunostained for perilipin 2 (NB110-40877SS, Bio-Techne) to evaluate lipid accumulation. For each tissue type, seven non-overlapping fields per section were randomly imaged using an Olympus microscope. Quantitative analysis was performed using ImageJ (NIH), and Sirius Red and immunohistochemical signals were normalized to the non-lipid area determined from H&E-stained sections to control for the extent of steatosis.

**RNA sequencing**

The paired-end reads that passed Illumina filters were filtered for reads aligning to tRNA, rRNA, adapter sequences, and spike-in controls. The reads were then aligned to the GRCm38.p4 reference genome and Gencode M9 annotations using STAR (v 2.6.1)[^46^](#_ENREF_46). DUST scores were calculated with PRINSEQ Lite (v 0.20.3) [^47^](#_ENREF_47) and low-complexity reads (DUST > 4) were removed from the BAM files. The alignment results were parsed via the SAMtools [^48^](#_ENREF_48) to generate SAM files. Read counts to each genomic feature were obtained with featureCounts (v 1.6.5) [^49^](#_ENREF_49). After removing absent features (zero counts in all samples), the raw counts were then imported to the R/Bioconductor package DESeq2 (v 1.24.0) [^50^](#_ENREF_50) to identify differentially expressed genes among samples. P-values for differential expression are calculated using the Wald test for differences between the base means of two conditions. These P-values are then adjusted for multiple test correction using the Benjamini-Hochberg algorithm. Genes that are consistently upregulated and downregulated were identified using linear regression. Gene set enrichment analysis was done using the ‘GseaPreranked’ method with the ‘classic’ scoring scheme at GSEA Software (v4.4.0). Rank files for each DE comparison of interest were generated by calculating π-value = -log10(p-adj) × LFC [^51^](#_ENREF_51).

**Electron microscopy**

Left ventricular (LV) samples collected as described above were prepared for transmission electron microscopy (TEM). Immediately following dissection, LV tissue fragments (~1–2 mm³) were fixed with 2% paraformaldehyde + 2.5% glutaraldehyde in 0.15 M sodium cacodylate buffer and further postfixed in 1% OsO4 in 0.15 M cacodylate buffer for 1hr on ice. The specimens were stained with 2% uranyl acetate for 1hr on ice, following graded dehydration in series of ethanol (50-100%) while remaining on ice. The cells were then subjected to one wash with 100% ethanol and two washes with acetone (15min each) and embedded with Durcupan (Sigma-Aldrich #44610-1EA). Sections were cut at 60 nm on a Leica UCT ultramicrotome, and picked up on 300 mesh copper grids. Sections were post-stained with 2% uranyl acetate for 5 minutes and Sato's lead stain (1% lead acetate, 1% Lead Nitrate, 1% Lead citrate and 2% sodium citrate in water) for 1 minute. Finally, the sections were observed at the UCSD Electron Microscopy Core (UCSD-CMM-EM Core, RRID:SCR_022039) using a Jeol 1400 plus operated at 80KeV and equipped with a bottom-mounted Gatan One View camera. Cristae score was calculated according to the scoring matrix as before [^45^](#_ENREF_45).

**Transthoracic Echocardiography**

Before echocardiography, a depilatory cream is applied to the anterior chest wall to remove hair. Mice are anesthetized with 5% isoflurane for 15 seconds, then maintained at 0.5% isoflurane throughout the echocardiographic examination. Small needle electrodes are inserted into one upper and one lower limb for simultaneous electrocardiogram (ECG) recording. Transthoracic echocardiography, including M-mode and 2-dimensional imaging, is performed using the FUJIFILM VisualSonics Inc. Vevo 2100 high-resolution ultrasound system with a linear transducer (32-55 MHz). Measurements of chamber dimensions and wall thickness are taken. Percentage fractional shortening (%FS) is used as an indicator of systolic cardiac function. Pulsed wave Doppler in the apical 4-chamber view is employed to acquire the ratio of peak velocities of early to late mitral inflow (E/A) and deceleration time (DT). Additionally, tissue Doppler imaging in the apical 4-chamber view allows for the measurement of mitral annular motion velocities, with E' and A' representing the peak mitral annular velocities during early and late filling, respectively. To ensure proper E and A wave separation, additional ketamine (50 mg/kg) is administered.

**Quantitative Real-Time PCR (qRT-PCR)**

Total RNA was extracted using RNeasy Mini columns (Qiagen, Valencia, CA), and reverse transcription was performed according to standard protocols. qRT-PCR was conducted using the QuantStudio 5 system (Applied Biosystems, Carlsbad, CA), and gene expression levels were normalized to *Hprt* using the ΔΔCt method. Primers were designed via PrimerBank (<https://pga.mgh.harvard.edu/primerbank/>). Data are presented either as fold change relative to the indicated control or as expression normalized to a housekeeping gene.

Following primers (mouse) were used:

**Metabolic Phenotyping**

Whole-body energy homeostasis was assessed using the Comprehensive Lab Animal Monitoring System (CLAMS; Columbus Instruments, OH). 6-months WD fed Male Foz/Foz (n:4) and WT (n:5) mice assessed for metabolic phenotyping. Mice were individually housed in metabolic chambers with ad libitum access to WD and water. After a 24-hour acclimation, continuous metabolic data were recorded over 48 hours under standard light/dark conditions. Parameters measured included VO₂, VCO₂, respiratory exchange ratio (RER), energy expenditure (EE), food and water intake, and locomotor activity (horizontal and vertical beam breaks). Data were acquired every 15 minutes and analyzed as light/dark phase-specific and 24-hour averages. EE was calculated using the Lusk equation based on VO₂ and VCO₂.

**Plasma Biochemistry**

Peripheral blood was collected from the inferior vena cava, and plasma was isolated using BD Microtainer tubes (cat. 365985, BD). ALT, ALP, cholesterol, total bilirubin, albumin, and BUN were measured using the Abaxis Mammalian Liver Profile system (catalog no#). Plasma triglycerides were quantified using the Triglyceride Reagent Set (236-60; Sekisui Diagnostics, PE, Canada). Plasma BNP levels were assessed using a mouse BNP ELISA Kit (NBP2-70011) following manufacturer protocols.

**Manuscript Preparation**

Some portions of the text were edited for clarity and conciseness using ChatGPT (OpenAI). All content was reviewed and verified for accuracy by the authors.

**Reporting and Study Design Details**

Outcome assessment was performed blinded to group allocation where feasible; histological scoring, morphometric image analyses, plasma biochemistry, and qRT-PCR were conducted using coded samples. Sample size (n = individual mice) is shown in the figures (each dot represents one animal) or stated in the figure legends; sample sizes were based on prior experience with this model, and no a priori sample size calculation was performed. No predefined inclusion or exclusion criteria were applied, and no animals or data points were excluded from analysis. To minimize potential confounders, animals from different groups were processed and measured in parallel and in mixed order whenever feasible; cage location was not treated as an experimental variable. No formal preregistration or publicly registered study protocol was completed prior to the study.

**SUPPLEMENTARY DATA FIGURES AND FIGURE LEGENDS**


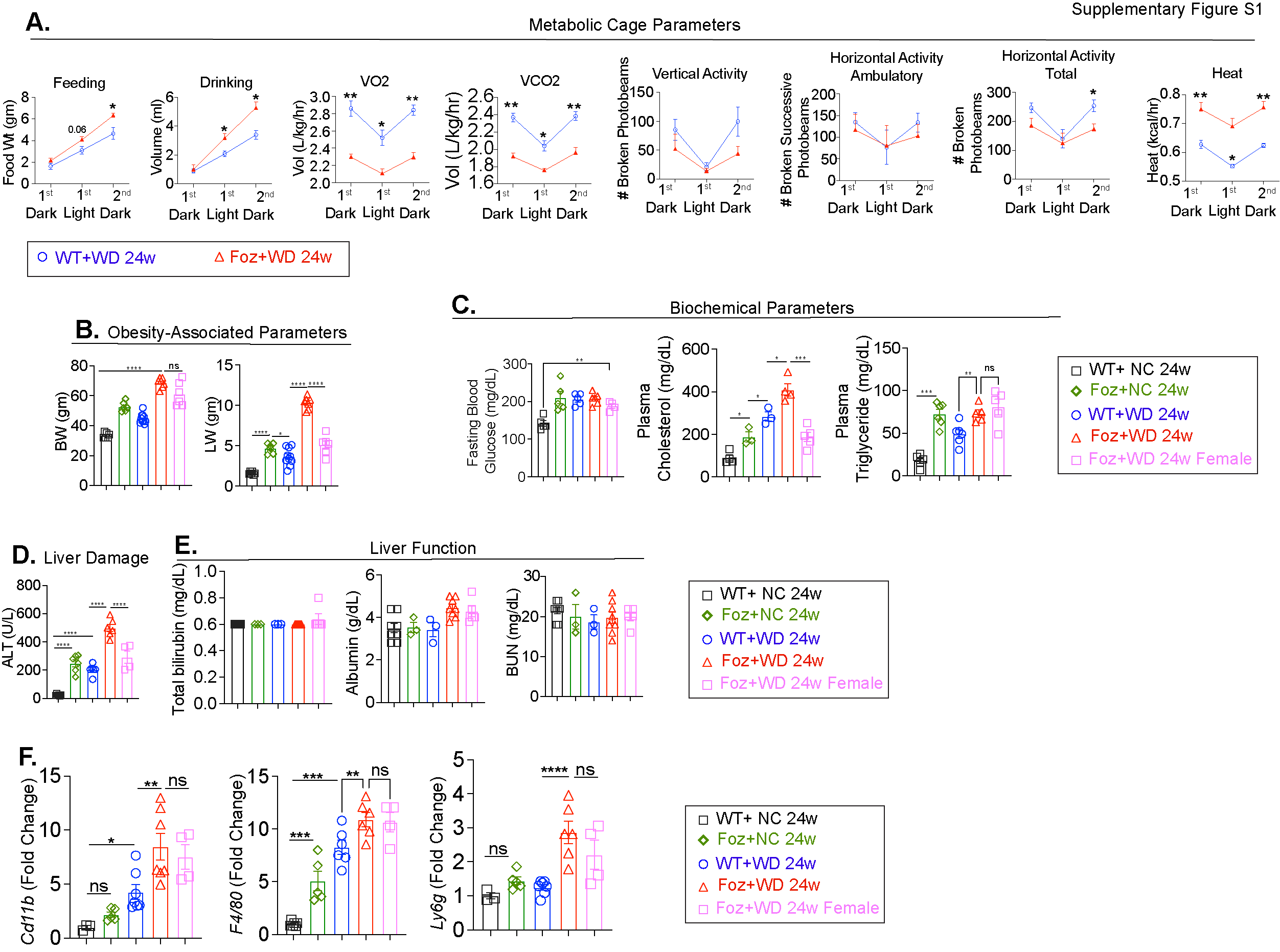


**Supplementary Fig. S1: A pre-clinical model of MetS**, **MASH and cardiometabolic dysfunction with mortality.** (A) WT+WD 24w and Foz+WD 24w mice were placed in instrumented metabolic cages individually (n=5 and 4 respectively) to monitor multiple metabolic parameters as indicated. (B-F) 6-8w old mice of indicated genotypes were either fed NC or WD for 24w. Liver and plasma were collected for subsequent analyses. (B) Body weight (BW) and liver weight (LW), across the indicated groups are plotted (Obesity-associated parameters). (C) Plasma fasting blood glucose, plasma cholesterol, plasma triglyceride levels are plotted (biochemical parameters). (D) Circulating alanine aminotransferase (ALT) levels reflecting liver injury. (E) Liver function markers, including total bilirubin, albumin, and blood urea nitrogen (BUN) are plotted. (F) Hepatic mRNA expression of immune cell markers *Cd11b* and *F4/80* (monocytes/macrophages), and *Ly6G* (neutrophils) were assessed by qRT-PCR, normalized to *Hprt* expression and plotted as fold change relative to healthy controls (WT+NC 24w). Data are presented as mean±SEM; One-way ANOVA; *p<0.05, **p<0.01, ***p<0.001, ****p<0.0001, ns=not significant.


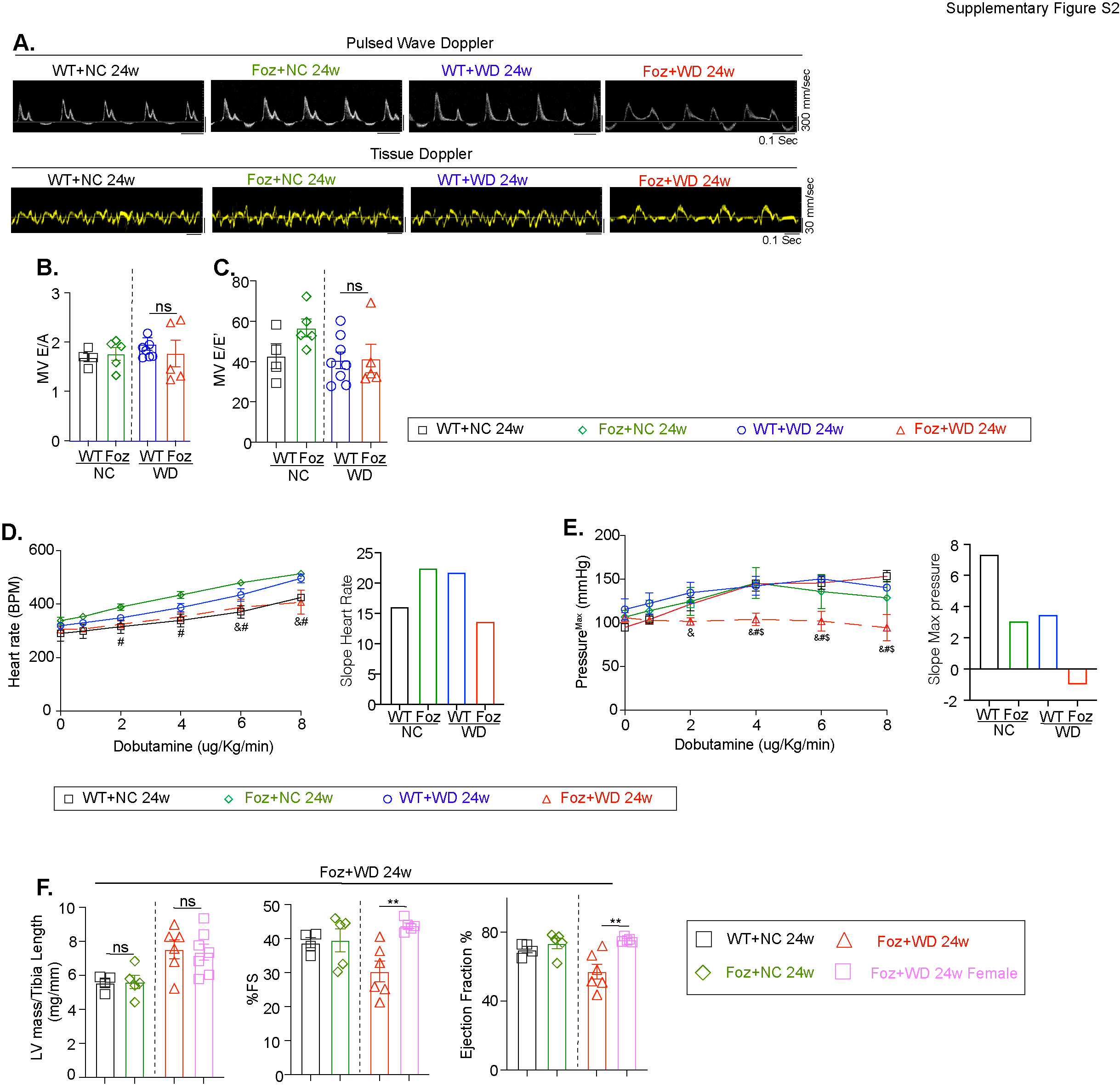


**Supplementary Fig. S2: Left ventricular dysfunction in MASLD.** (A) Representative Doppler echocardiography images including pulse-wave (PW) Doppler and tissue Doppler echocardiography (TDE) used to assess LV filling and myocardial relaxation. (B) Mitral valve early-to-late filling velocity ratio (MV E/A). (C) Mitral valve early filling velocity to early diastolic mitral annular velocity ratio (MV E/E’), an indicator of LV filling pressure. (D-E) Hemodynamic indices of systolic and diastolic function under dobutamine stress. (D) Heart rate (BPM) changes, and (E) Maximum LV pressure (pressure^max^) across indicated groups are plotted along with the slope of the respective curves. (F) Female Foz+WD 24w echocardiographic parameters measured and plotted with the other groups (males) for comparison as shown in Figure 2B, I, and J. Data are presented as mean±SEM; One-way/Two-way ANOVA. **p < 0.01, ns=not significant. For (D-E) symbols, &, #, $, indicate P < 0.05 for the respective comparisons as indicated. & : Foz+WD 24w vs WT+WD 24w # : Foz+WD 24w vs Foz+NC 24w $ : Foz+WD 24w vs WT+NC 24w


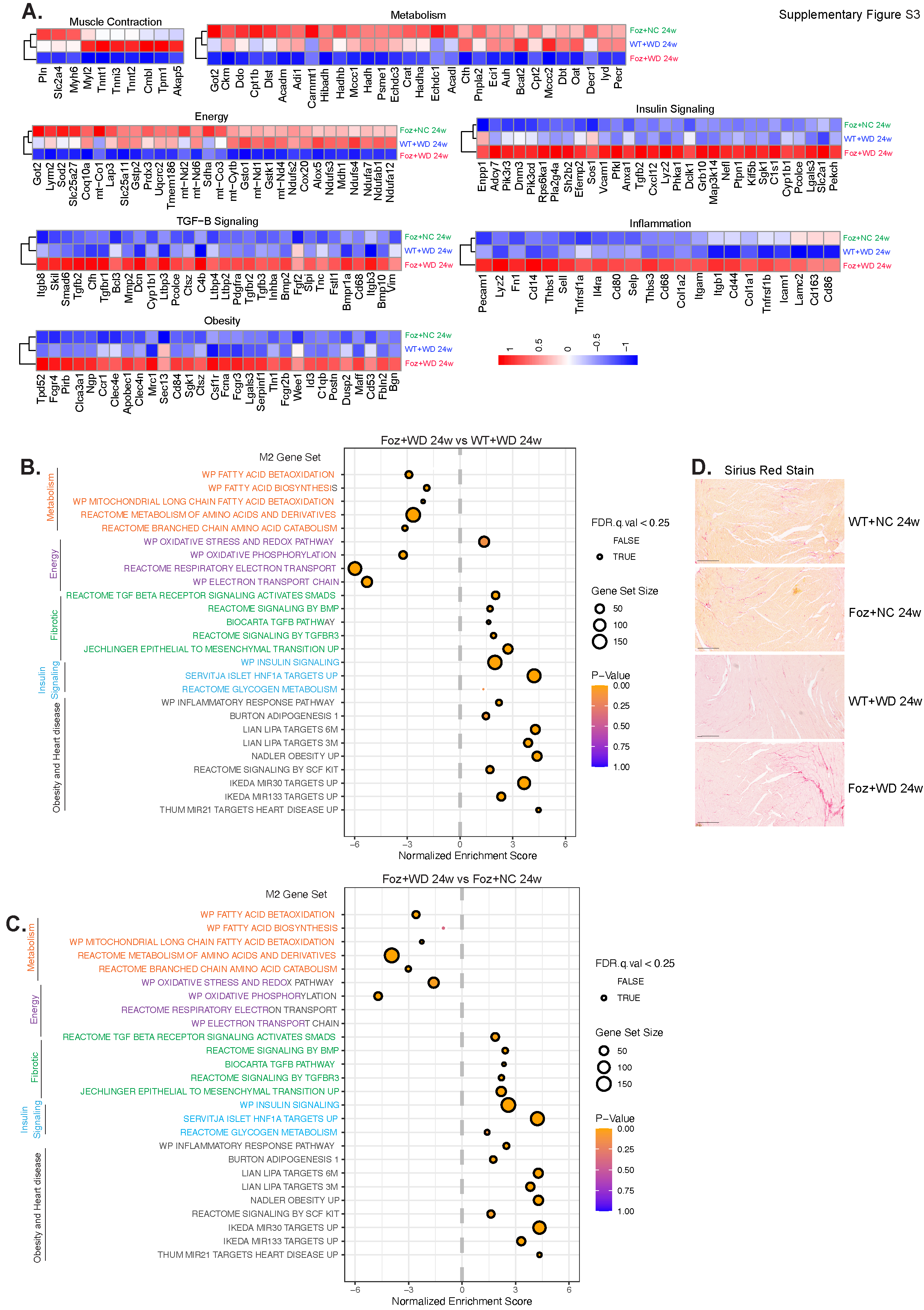


**Supplementary Fig. S3: Transcriptome reveals key pathways associated with LV dysfunction.** (A) Heatmap showing the relative expression of genes from analysis in FIGURE 3C and 3D. (B-C) Scatter plot illustrating GSEA between (B) Foz+WD 24w vs WT+WD 24w and (C) Foz+WD 24w vs Foz+NC 24w, using the M2 Curated gene set. The size of each dot represents the gene set size, dark circles indicate significant enrichment based on FDR q-val < 0.25, and the color corresponds to the p-value. (D) Representative Sirius red (SR) stained mouse heart sections of the indicated groups.


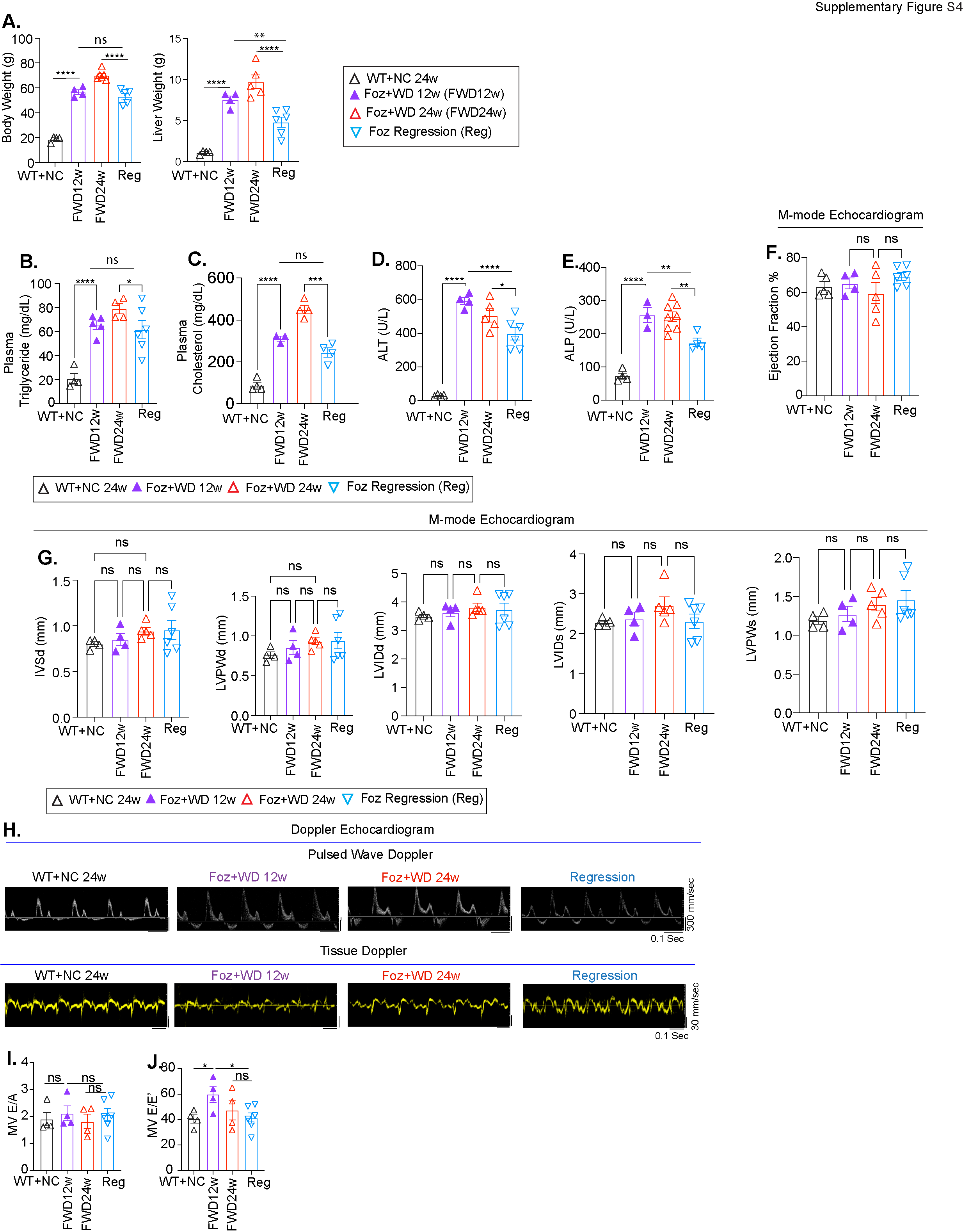


**Supplementary Fig. S4: Effect of MASH-fibrosis regression on cardiac remodeling.** (A) Body weight and liver weight measurements in the indicated groups. (B-E) Plasma metabolic and liver injury markers were measured. (B) Triglycerides; (C) cholesterol; (D) alanine aminotransferase (ALT); (E) alkaline phosphatase (ALP). (F, G) M-mode echocardiographic measurements of LV structure: (F) ejection fraction (EF); (G) interventricular septum thickness at diastole (IVSd), posterior wall thickness at diastole (LVPWd), LV internal diameter at diastole (LVIDd), internal diameter at systole (LVIDs), and posterior wall thickness at systole (LVPWs). (H) Representative Doppler echocardiographic images, including pulsed-wave (PW) Doppler and tissue Doppler imaging (TDI), used to assess LV filling and myocardial relaxation. (I-J) Diastolic function indices were measured and plotted: (I) Mitral valve early (E) to atrial (A) wave velocity ratio (MV E/A) and (J) Mitral valve early (E) to early mitral annular velocity (E') ratio (MV E/E'). Data are presented as mean±SEM; One-way ANOVA; *p<0.05, **p<0.01, ***p<0.001, ****p<0.0001, ns=not significant.


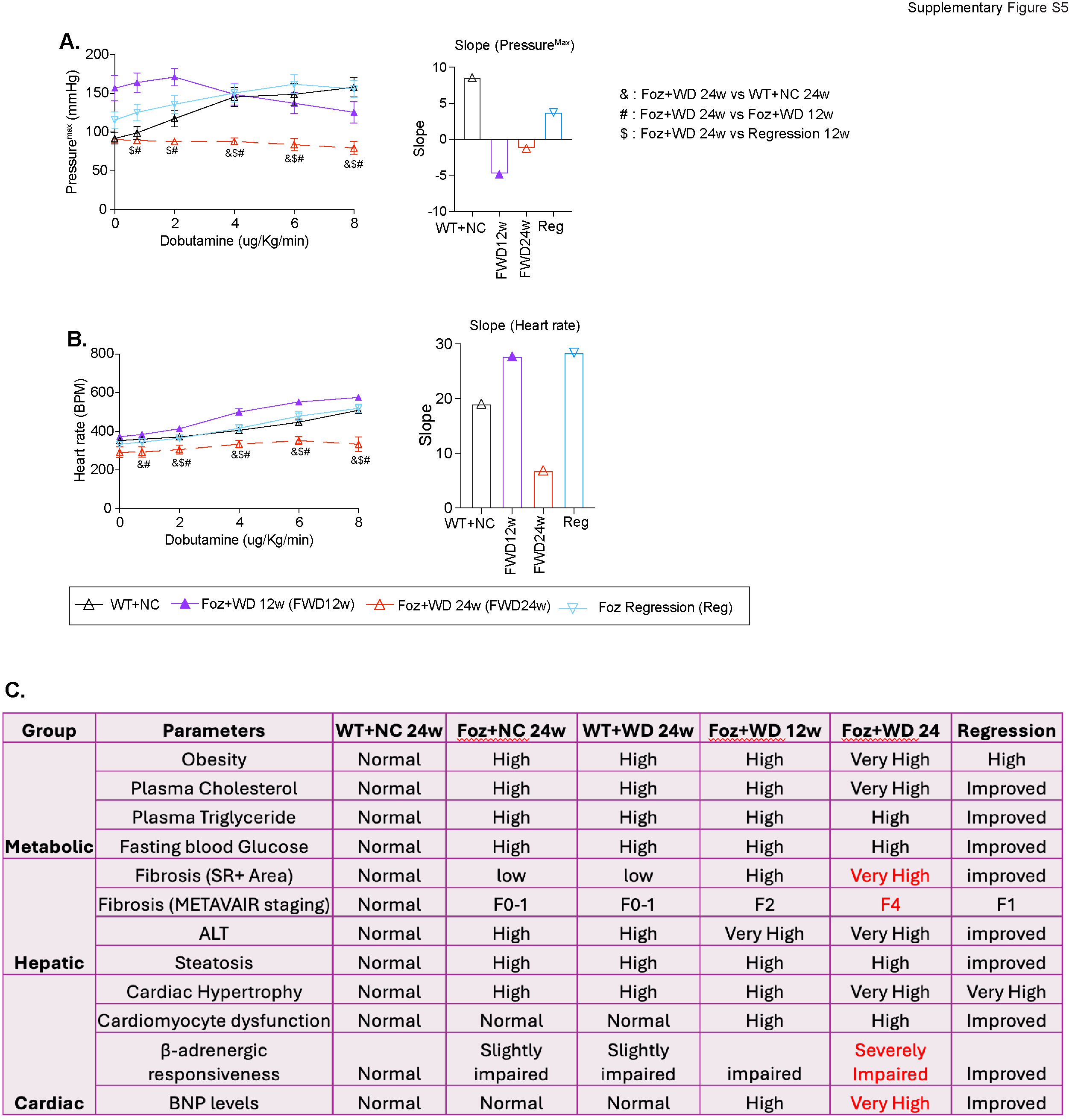


**Supplementary Fig. S5:** **Dietary intervention improves** **LV dysfunction and overall survival.** (A-B) Hemodynamic parameters under dobutamine stress: (A) maximum left ventricular pressure (Pressure^max^); (B) heart rate (beats per minute, BPM), along with corresponding slopes of response curves across the indicated groups. Two-way ANOVA. Symbols, &, # and $ indicate P < 0.05 for the respective comparisons. & : Foz+WD 24w vs WT+NC 24w, # : Foz+WD 24w vs Foz+WD 12w, $ : Foz+WD 24w vs Regression 12w (C) Summary table showing metabolic, hepatic, and cardiac phenotypes across different experimental groups.


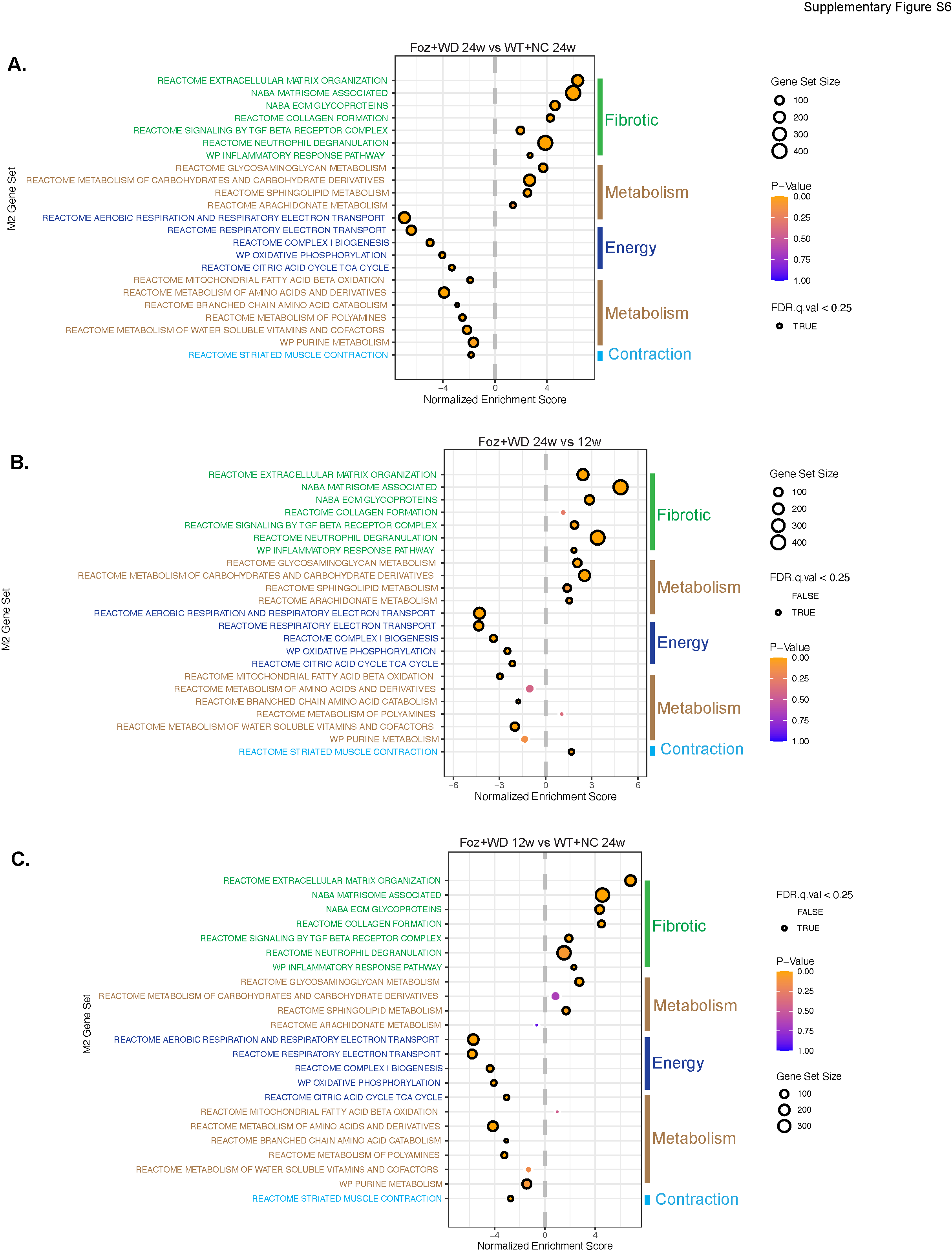


**Supplementary Fig. S6: LV remodeling pathways and gene expression changes.** (A-C) Bubble scatter plot illustrating gene set enrichment analysis (GSEA) using the M2 Curated gene set to compare (A) Foz+WD 24w vs WT+NC 24w, (B) Foz+WD 24w vs Foz+WD 12w (C) Foz+WD 12w vs WT+NC 24w. The size of each dot represents the gene set size, dark circles indicate significant enrichment based on FDR q-val< 0.25, and the color corresponds to the p-value.


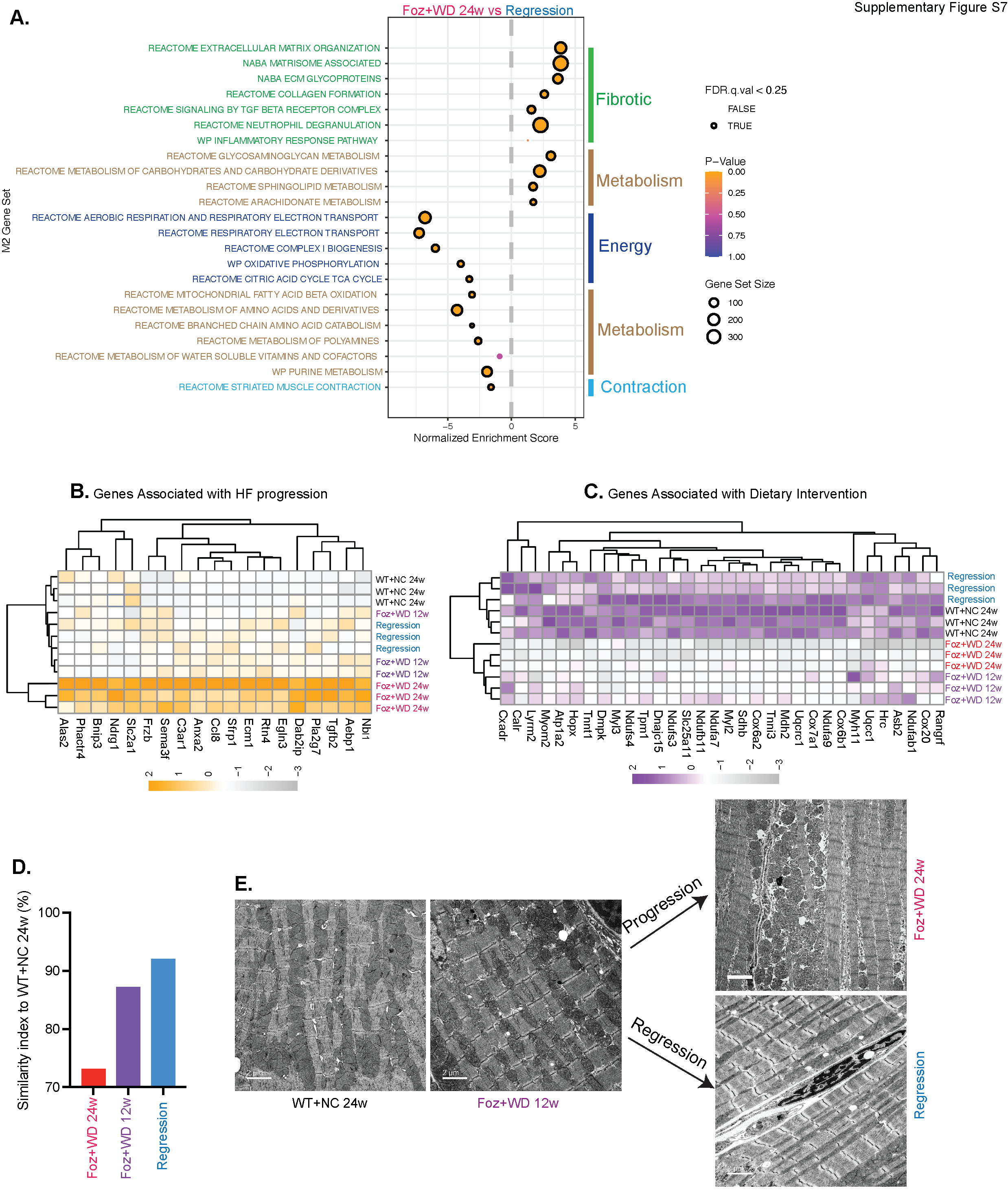


**Supplementary Fig. S7: Changes in LV gene expression during disease regression**. (A) GSEA comparing regression vs Foz+WD 24w groups using the M2 curated gene set. Each dot represents an individual gene set, with size indicating the number of genes, color representing P value, and dark circles denoting significantly enriched pathways (FDR q < 0.25). (B-C) Heatmap showing the genes (Z-score) driving (B) the heart failure associated and (C) regression-associated pathways (related to Figure 7B, C). (D) Cosine similarity index (%) (based on Figure 7D) of LV transcriptomes from Foz+WD 24w, Foz+WD 12w, and regression (WD→chow) groups compared to healthy WT+NC 24w controls. Foz+WD 24w LV showed the lowest similarity, reflecting advanced disease-associated transcriptional reprogramming. In contrast, Foz+WD 12w mice exhibited intermediate similarity, and regression mice displayed the highest similarity to healthy transcriptomes, indicating substantial molecular restoration following dietary intervention. (E) Representative TEM images (lower magnification of Figure 7F) showing progressive mitochondrial damage with worsening fibrosis and HFpEF in Foz/Foz mice, and restoration of ultrastructural integrity after fibrosis resolution in the dietary intervention (regression) group.

**References:**

[1] Dobin A, Davis CA, Schlesinger F, Drenkow J, Zaleski C, Jha S, et al. STAR: ultrafast universal RNA-seq aligner. Bioinformatics. 2013;29:15-21.

[2] Schmieder R, Edwards R. Quality control and preprocessing of metagenomic datasets. Bioinformatics. 2011;27:863-4.

[3] Li H, Handsaker B, Wysoker A, Fennell T, Ruan J, Homer N, et al. The Sequence Alignment/Map format and SAMtools. Bioinformatics. 2009;25:2078-9.

[4] Liao Y, Smyth GK, Shi W. featureCounts: an efficient general purpose program for assigning sequence reads to genomic features. Bioinformatics. 2014;30:923-30.

[5] Love MI, Huber W, Anders S. Moderated estimation of fold change and dispersion for RNA-seq data with DESeq2. Genome Biol. 2014;15:550.

[6] Xiao Y, Hsiao TH, Suresh U, Chen HI, Wu X, Wolf SE, et al. A novel significance score for gene selection and ranking. Bioinformatics. 2014;30:801-7.

[7] Hsieh CC, Li CY, Hsu CH, Chen HL, Chen YH, Liu YP, et al. Mitochondrial protection by simvastatin against angiotensin II-mediated heart failure. Br J Pharmacol. 2019;176:3791-804.
